# *In vitro* derivation of megakaryocytes and platelets from induced pluripotent stem cells: developmental foundations and translational perspectives

**DOI:** 10.3389/fimmu.2025.1720048

**Published:** 2025-12-09

**Authors:** Maciej Mazurek, Wojciech Młynarski, Dawid P. Grzela

**Affiliations:** 1 Department of Pediatrics, Oncology and Hematology, Medical University of Lodz, Lodz, Poland; 2 Institute of Medical Biology, Polish Academy of Sciences, Lodz, Poland

**Keywords:** *in vitro* hematopoiesis, induced pluripotent stem cells (iPSCs), platelet biogenesis, megakaryocyte differentiation, cell-based transfusion therapy

## Abstract

For centuries, hematologists have strived to develop increasingly sophisticated systems and therapeutic protocols for replenishing the blood. However, demographic shifts have led to a growing demand for blood-derived products, and the number of eligible donors continues to decline, raising concerns regarding the future availability and cost-effectiveness of transfusion therapies. Advances in our understanding of molecular hematopoiesis, coupled with the development of precise gene-editing tools such as CRISPR/Cas9 and the advent of induced pluripotent stem cell (iPSCs) technology, have opened new avenues for the generation of functional blood components *in vitro*. The ability to reprogram somatic cells into pluripotent states offers a virtually unlimited and ethically acceptable source of patient-specific or universal donor-compatible cells for both research and therapeutic applications. This review summarizes the current strategies for the *in vitro* generation of megakaryocytes and functional platelets from iPSCs and outlines the developmental foundations of primitive and definitive hematopoiesis that underpin these efforts. Furthermore, we emphasize strategies aimed at improving maturation and yield, along with emerging approaches in HLA editing and immune tolerance designed to overcome alloimmune barriers in transfusion medicine.

## 1 Introduction: historical perspectives on platelets and transfusion medicine

Over the years, research on the bone marrow has led to revolutionary discoveries regarding the morphological components of blood. In 1882, physician Bizzozero described the third most numerous component, platelets, which have a disc-shaped structure and a diameter three times smaller than that of red blood cells. Until then, they had been considered fragments of disintegrated red blood cell elements, leukocytes, or even microbes. However, Bizzozero meticulously detailed its role in the clotting process and observed the recruitment of white blood cells to form platelet aggregates. This allowed researchers to link them to the pathogenesis of thrombosis and, a few years later, to atherosclerosis and coagulation disorders (1–3).

Shortly thereafter, in 1906, the introduction of a new staining technique by James Wright revealed that platelet granules stained identically to megakaryocyte granules, confirming that platelets originate by budding from giant bone marrow cells (2). In 1910, it was determined that platelet deficiency, defined as below 30 000/µL, was associated with spontaneous hemorrhage, which enabled the description of the pathogenesis of chronic bleeding linked to thrombocytopenia. In the same year, the first platelet transfusion was performed. Subsequent research confirmed that platelet production must be regulated by specific stimuli, with thrombopoietin identified as a key factor (4).

Modern medicine uses platelet transfusion as a therapy for the treatment of conditions associated with hemorrhage, both internal and external, such as hematological disorders and the effects of anticancer therapy. Platelet transfusions are used, among other things, to prevent severe thrombocytopenia resulting from anticancer therapies, congenital platelet deficiencies (e.g., mutations in *ETV6*, *RUNX1*, *ANKRD26*), and to treat profuse hemorrhage after extensive trauma. Moreover, demographic trends in developed countries are projected to significantly reduce the number of eligible donors, while the clinical need for platelet transfusions continues to increase with an aging population (5, 6).

One of the major challenges in platelet transfusion therapy is alloimmunization, a process in which the recipient’s immune system reacts to foreign antigens found on donor platelets. This reaction is driven by antibodies that specifically target human leukocyte antigen (HLA) class I molecules and human platelet antigens (HPA) located on the surface of platelets (7, 8). Patients, particularly those undergoing treatment for hematological or oncological conditions, who receive multiple transfusions, are prone to sensitization. This sensitization results in the generation of IgG anti-HLA and anti-HPA antibodies, which hasten the removal of platelets through processes like Fc receptor-mediated phagocytosis and complement activation. The clinical outcome of this immune response is platelet refractoriness (PTR), a condition where transfused platelets do not effectively raise blood cell counts or provide sufficient hemostatic protection (9–11).

Even with the implementation of leukoreduced blood products and HLA-matched donors, alloimmune refractoriness still impacts 30–70% of patients who require frequent transfusions, often resulting in a lack of compatible donor options (12). The significant diversity of HLA alleles, the scarcity of well-typed donors, and the quick expiration of stored platelet concentrates further intensify this issue. Consequently, alloimmunization is not only a complication related to transfusions but also a significant obstacle for maintaining sustainable platelet supply systems. These immunological constraints have become a key driver exploring hypoimmunogenic and universal platelet sources, especially those originating from pluripotent stem cells (13, 14). These approaches enable the generation of autologous or gene-edited platelet progenitors, offering a solution to HLA incompatibility and minimizing immune-mediated destruction. By engineering these cells to lack immunogenic determinants, such as through β2-microglobulin silencing or selective HLA expression modulation, a consistent, renewable, and potentially alloimmune-neutral platelet source for transfusion medicine can be developed.

In 2006, a groundbreaking discovery was made: the induction of pluripotent stem cells from somatic cells (15). The discovery and development of this method has allowed for large-scale, economically viable, and ethically viable use of iPSCs in human biology research, disease modeling, and regenerative medicine (16–19). Rapid advancements in this field have found particular applications in experimental hematology, where efforts are underway to obtain specific blood components by differentiating iPSCs under *in vitro* conditions (20–22).

Discoveries made over the past 150 years in hematology, together with the dynamic progress of cellular engineering, provide the basis for a new era of therapies in which platelet production *in vitro* is no longer a theoretical concept but an emerging clinical reality. This review delves into the biological and technological foundations that facilitate the transformation of induced pluripotent stem cells into megakaryocytes and platelets, with a focus on the molecular mechanisms that differentiate primitive from definitive hematopoiesis. In addition to mapping out these developmental routes, we emphasize current initiatives to enhance the functional maturation and production of platelets generated *in vitro*. We also explore recent advancements in HLA-editing and immune-tolerance engineering, which aim to address alloimmune challenges in transfusion medicine.

## 2 Hematopoiesis: developmental origins and lineage specification

### 2.1 Primitive hematopoiesis: yolk sac–derived blood progenitors

A critical aspect of embryonic hematopoiesis is the proper development of erythrocytes, megakaryocytes, and macrophages during primitive hematopoiesis, which is limited to these three cell types. Disruption of this process leads to disorders that can result in embryonic death (23). Within the first two weeks of gestation following the initiation of gastrulation, clusters of extraembryonic mesodermal progenitors - called “blood islands” - form in the yolk sac. The initial phase of primitive hematopoiesis involves differentiation of mesodermal cells from blood islands into bipotential primitive megakaryocyte-erythroid progenitors (pMEPs), endothelial cells, and unipotential macrophages within the yolk sac (24–26). Macrophage maturation begins later than that of other progenitors, and their differentiation occurs directly, bypassing monocyte intermediacy (27, 28). In mice, primitive MEPs are observed along with primitive megakaryocytes and erythroid progenitors as early as embryonic day 7 (E7.5). Furthermore, by E9.5, the first cells expressing GP1bβ (CD42c) were observed on their surface (29).

Blood islands exhibit a heterogeneous structure: cells located centrally give rise to primitive erythroid and other myeloid progenitors, whereas peripheral cells differentiate into endothelial cells, which are the precursors of blood vessels. The formation of these channels is crucial for further expansion of progenitor cells and continued development of embryonic hematopoiesis (23, 30). With the onset of differentiation and proliferation of primitive progenitors, another population of erythro-myeloid progenitors (EMPs), highly proliferative multipotent colony-forming cells (HPP-CFCs), begins to emerge, serving as a bridge between primitive and definitive hematopoiesis. At the same time, another crucial population appears during immune system development: lymphoid-primed multipotent progenitors (LMPPs), which give rise to B and T cell progenitors (31).

The fusion of blood islands with the vascular endothelium leads to the budding of erythroid and myeloid progenitors into the vessel lumen via a conserved process called the endothelial-to-hematopoietic transition (EHT) (32, 33). Subsequent colonization of the fetal liver is referred to as the second wave of primitive hematopoiesis or the transitional (pro-definitive) wave. Primitive erythropoiesis precursors are transient, and following liver colonization they become undetectable in the yolk sac during later stages of development (34). On day 23 of human gestation, upon reaching the liver, EMP cells begin to differentiate into multiple blood cell types, including enucleated erythrocytes, megakaryocytes, and monocytes (35). These monocytes infiltrate developing tissues via circulation and initiate the formation of tissue-resident macrophage niches (27).

In the mouse model, between E10.5 and E12.5, an increase in highly reticulated and large platelets was observed in the bloodstream, accompanied by a decrease in primitive MKPs in the yolk sac and a growing number of progenitors in the liver. From E13.5 onward, smaller platelets appear, which may indicate their release into the circulation from more mature (non-primitive) forms of megakaryocytes (29).

In humans, this process has not been well characterized. However, based on descriptions of primitive hematopoiesis in mice and histopathological studies of the human fetal liver, it is believed that between the 3rd and 4th week of gestation, CD34^+^ and CD45^+^ cells are present in the liver. This occurs even before the appearance of hematopoietic stem cells (HSCs), which are detectable only around the 5th to 6th week (28).

### 2.2 Definitive hematopoiesis: emergence of HSCs and megakaryopoiesis

The third wave involves the replacement of primitive hematopoiesis with definitive hematopoiesis, with a key aspect being the emergence of hematopoietic stem cells (HSCs) and the relocation of the hematopoietic process to the fetal organism. As previously described, the first stage of definitive hematopoiesis (pro-definitive) involves the emergence of erythromyeloid progenitors (EMPs) that reach the liver via the bloodstream, thereby initiating the first wave of definitive hematopoiesis (23).

As a result of the endothelial-to-hematopoietic transition that takes place in the 4th to 5th week of pregnancy, HSCs start to develop from clusters of hemogenic endothelial cells found in the fetal dorsal aorta. This process also occurs at other key sites such as the umbilical and vitelline arteries of the embryonic arterial system. In mouse fetuses, a similar phenomenon is observed between embryonic day 9.5 (E9.5) and E11.5 in the aorta–gonad–mesonephros (AGM) region (36). These cells give rise to all hematopoietic progenitors, including long-term reconstituting hematopoietic stem cells (LT-HSCs), enucleated red blood cells, myeloid cells, lymphocytes, and definitive megakaryocytes (37). From the 7th week of gestation, HSCs derived from the hemogenic endothelium colonize the fetal liver, where they continue to mature and differentiate (38). At this stage, the liver becomes the primary hematopoietic organ until this function is assumed by the bone marrow. Around the 20th week of gestation, HSCs also colonize the spleen (24). HSCs settle in the developing bone marrow, which gradually becomes the main hematopoietic organ (39). By the end of the third trimester, structural changes in the bone marrow lead to the formation of marrow cavities that serve as reservoirs for HSCs (40).

*RUNX1* is considered one of the key genes regulating both primitive and definitive hematopoiesis. Expression studies have demonstrated high levels of RUNX1 in HSCs as well as in endothelial cells of the yolk sac, fetal vitelline, umbilical arteries, and AGM region, suggesting its essential role in the EHT process (41). Germline mutations in *RUNX1* are associated with familial platelet disorder and myeloid malignancy (FPDMM, OMIM:601399) (42).

**Figure 1:**
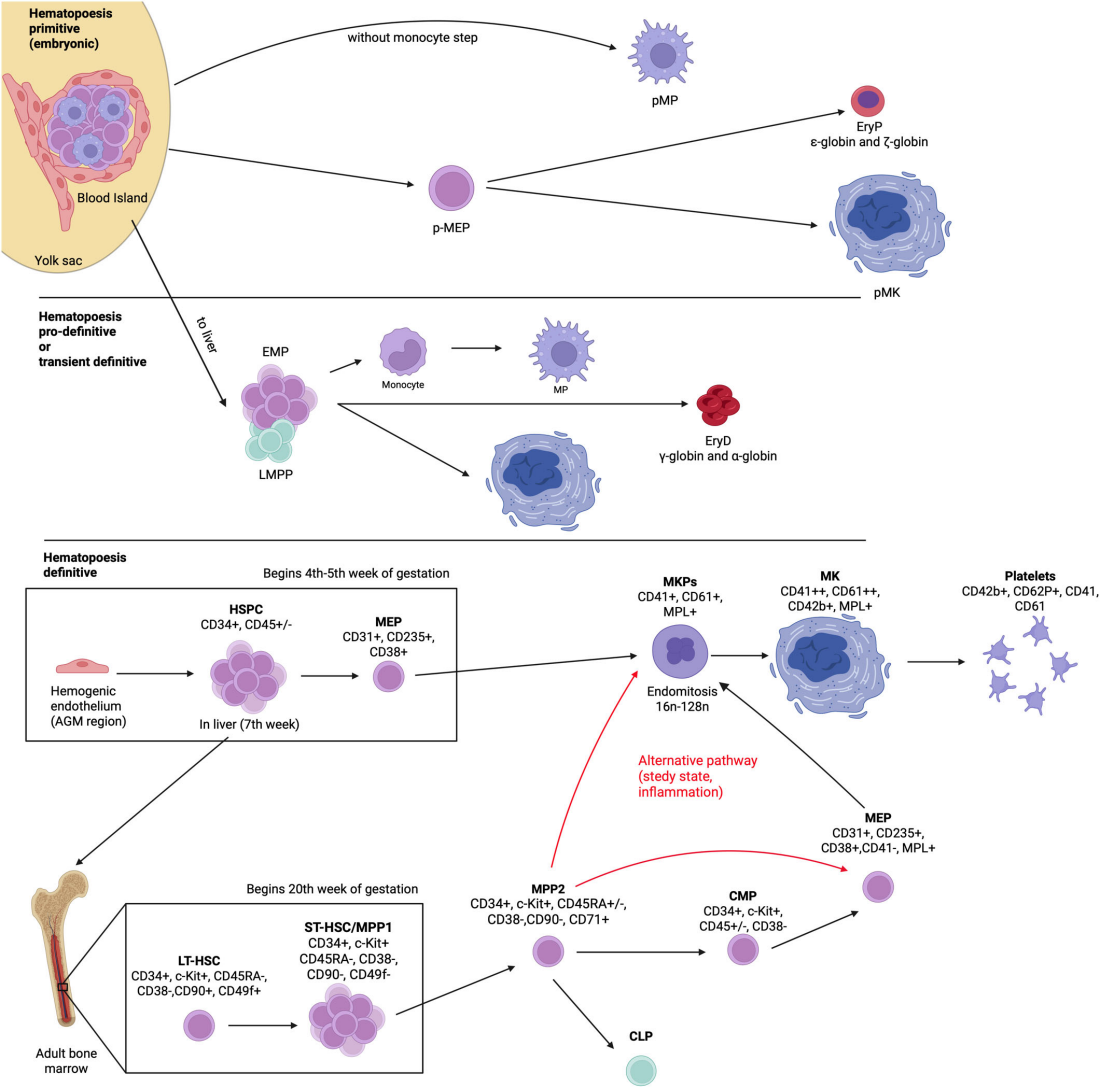
Overview of megakaryopoiesis across developmental hematopoiesis. The diagram illustrates the differentiation trajectory from yolk sac–derived primitive progenitors (pMEP, EMP) to definitive megakaryocytes in the adult bone marrow, including the intermediate stages in the fetal liver. Surface marker expression (CD41, CD42b, and CD61) and proliferative features such as endomitosis are indicated. Created with BioRender.com.

In definitive hematopoiesis within the adult bone marrow, lineage specification follows a hierarchical structure and is associated with a gradual loss of differentiation potential (**Figure 1**). As in primitive and pro-definitive hematopoiesis, megakaryopoiesis is tightly linked to erythropoiesis, with thrombopoietin (TPO) and erythropoietin (EPO) acting as key regulators. According to the classical model of hematopoiesis, LT-HSCs give rise to short-term hematopoietic stem cells (ST-HSCs) that subsequently generate multipotent progenitors (MPPs). Compared to HSCs, MPPs lack the self-renewal capacity (43). The first lineage bifurcation yields two oligopotent progenitor types: common myeloid progenitors (CMPs) and common lymphoid progenitors (CLPs) (44). Differentiation from CMPs leads to the formation of bipotent megakaryocyte–erythroid progenitors (MEPs), which subsequently transition to unipotent megakaryocyte progenitors (MkPs) (45, 46).

While the classical hematopoietic model remains a cornerstone for understanding lineage differentiation, recent studies have revealed substantial heterogeneity within the HSC compartment (47–49). Similarly, MPPs do not constitute a uniform population but instead comprise multiple subtypes, with MPP type 2 showing the highest plasticity toward megakaryocyte differentiation. These cells can follow the “classical” pathway through CMPs or differentiate directly into MEPs or MkPs, bypassing the CMP stage (50, 51).

Recent transplantation and lineage-tracing studies in mice have identified a subset of HSCs expressing von Willebrand factor (HSC vWF^+^), which retains multipotency but displays differentiation bias toward the megakaryocyte lineage. Interestingly, HSC vWF^+^ cells can give rise to vWF^-^ progeny, but not vice versa, suggesting that vWF^+^ cells occupy a higher hierarchical position (52, 53). These findings highlight a much greater plasticity in the hematopoietic system than previously assumed and underscore the diverse pathways available for megakaryocyte lineage commitment.

## 3 Platelet biology and thrombopoiesis

### 3.1 Structural and functional properties of platelets

Platelets are the second most abundant morphotic component, following RBCs. After being released into the bloodstream, they circulate for a short period of 7–10 days. Under normal physiological conditions, they are non-nucleated cell fragments ranging in size from 2 to 5 µm (54). The lack of a cell nucleus limits the platelet responsiveness to both intra- and extracellular stimuli. Platelets contain various RNA species inherited from precursor megakaryocytes and granules packed with bioactive mediators such as von Willebrand factor, vascular endothelial growth factor (VEGF), transforming growth factor-β (TGF-β), and platelet-derived growth factor (PDGF), which collectively modulate cellular responses (55).

Despite their simple structure, blood platelets do not constitute a homogeneous population. They vary in size, with smaller platelets transporting more transcripts of inflammatory response factors than larger subpopulations (56). Moreover, under certain conditions, the inflammatory response can be indirectly modulated by megakaryocytes through blood platelets. This occurs during antiviral responses when platelets contain increased levels of antiviral proteins synthesized during thrombopoiesis (57). Additionally, blood platelets modulate the immune response and are involved in the regulation of angiogenesis (7, 58, 59).

Although platelet biology is relatively well-characterized, the processes of megakaryocyte differentiation and maturation remain poorly understood. Megakaryocytes are large cells with diameters ranging from 50 to 100 µm, constituting a small fraction of the bone marrow (approximately 0.05%) (60). It is also known that the final maturation process, which is necessary for platelet production, is multi-step. For this purpose, the cell undergoes a series of internal mitotic divisions without the ultimate cell division (endomitosis). This is a specific type of cell cycle, in which only the DNA synthesis phase (S phase) lasts for the same amount of time as in other human cells (approximately 6–7 h), while the G1 and G2 phases are abbreviated. The course of endomitosis from prophase to anaphase A is similar to that of classical mitotic division, however, the cell does not progress to further phases of this process. The spindle apparatus does not separate and the individual sister chromatids are not transported to the cell poles. Subsequently, the nuclear envelope is reformed, resulting in a polyploid nucleus. During this division, telophase and cytokinesis are bypassed and the cells immediately enter the G1 phase of the cell cycle (61).

This process gives rise to polyploid megakaryocytes with 16–128 cell nuclei. The cell membrane also plays a crucial role in thrombopoiesis. As megakaryocytes mature, they develop an extensive demarcation membrane system (DMS), that invaginates into the cytoplasm and delineates future proplatelet territories (62). Ultimately, cytoplasmic extensions are formed, protruding into the lumen of blood vessels. Shear forces from flowing blood and high concentrations of sphingosine-1-phosphate assist in detaching and releasing cytoplasm into the bloodstream, thereby forming anucleate platelet fragments (63). The second proposed concept is extracellular interaction, which is indirectly mediated by interleukin 1α in acute inflammatory states. As a result of cytokine interactions with megakaryocytes in the bone marrow, there is a rapid release of large amounts of platelets (64). The shear-dependent mechanism represents the primary physiological route of proplatelet elongation and platelet release within the bone marrow and lung microcirculation (65). In contrast, the IL-1α-driven pathway reflects an acute stress-induced response, enabling rapid, rupture-mediated platelet release during inflammatory or high-demand states, and therefore does not replace but complements the basal hemodynamic mechanism (64). Regardless of the mechanism of thrombopoiesis induction, significant transformations in the cytoskeleton, mainly in microtubules, play a crucial role in this process (66). Research in mice confirmed that the most active site of thrombopoiesis is the lungs, accounting for over half of the total number of platelets released in mouse models (67). However, this phenomenon has not yet been confirmed in humans.

### 3.2 Primitive vs. definitive megakaryocytes: developmental and functional differences

Megakaryocytes are created in early hematopoietic waves. Like other blood components produced in this process, primitive megakaryocytes differ from those produced during definitive hematopoiesis. Primitive megakaryocytes are smaller than their definitive counterparts and exhibit lower proliferative activity. Their endomitotic activity is also reduced, which affects the number of platelets produced (68). Studies on specific genes related to megakaryocytes also revealed significant differences in their expression (69). Although the expression and function of genes closely related to megakaryocyte maturation, such as *GATA1*, *FOG1*, *NFE2*, and *RUNX1*, are well understood, their expression is reduced or even completely suppressed during the early stages of embryogenesis. Conversely, factors like LIN28B and MYC homologs (C-Myc, N-Myc), which are linked to higher proliferation and growth rates, show high expression. The regulation of these genes is tightly controlled by microRNAs (for instance, miR-145 regulating *MYC* and miR-181a targeting *LIN28B*) and is associated with specific ontogeny stages (69). Furthermore, increased *MYC* expression in later stages of megakaryocyte maturation may be linked to the suppression of *GATA1* and *NFE2*, contributing to impaired terminal differentiation (70). As ontogeny progresses, *LIN28B* and *MYC* family members become downregulated, while the previously mentioned maturation-related transcription factors and those associated with cell-cycle control, polyploidization, and hemostasis (such as *STAT5A*, *ETS2*, *CXCR4*, and *VWF*) are upregulated. Additionally, there is an increase in the expression of characteristic megakaryocytic surface markers like CD41, CD42b, and CD49b (71, 72).

Platelets obtained from primitive megakaryocyte lineages are larger and have a more reticulated morphology than those generated during definitive hematopoiesis (29, 73). Moreover, the microtubular structure is less developed, and there are fewer alpha granules that contain growth factors (74, 75), indicating a potentially lower secretory activity of platelets derived from primitive hematopoiesis.

Compared to adult groups, aggregometry tests indicate that the reactivity of platelets from umbilical cord blood is reduced depending on the agonist used. This is likely due to the inhibition of key signaling pathways essential for endogenous platelet activation, such as the significantly reduced response of the thromboxane synthesis pathway while maintaining a similar number of receptors, which suggests inhibition of downstream signaling (76). Additionally, studies have confirmed a quantitatively lower presence of α_2_-adrenergic receptors, which correlates with a reduced response to epinephrine (77). However, the results of whole-blood tests appear inconsistent, indicating that the coagulation time of newborns/umbilical cord blood is the same or even shorter. These findings may be associated with high levels of von Willebrand factor and elevated hematocrit (78, 79).

## 4 *In vitro* generation of megakaryocytes from induced pluripotent stem cells

### 4.1 Reprogramming strategies and immune editing

The development of somatic cell reprogramming into iPSCs has revolutionized regenerative medicine and disease modeling. This technology allows the creation of patient-specific pluripotent cells. It overcomes many ethical and technical issues related to embryonic stem cells (ESCs) (80). Before reprogramming methods were introduced, ESCs were the main source of pluripotent cells. While ESCs have several benefits, many of which iPSCs also share, their use is still controversial because it involves destroying human embryos during their derivation. iPS cells are generated by reprogramming somatic cells through transient overexpression of defined transcription factors, most commonly OCT3/4, SOX2, KLF4, and c-MYC. This process erases lineage-specific epigenetic marks and reactivates networks of genes associated with pluripotency. As a result, it restores the ability to self-renew and differentiate into all three germ layers. Early methods for inducing pluripotency used vectors that integrated into the host genome. While these systems are effective, they present risks, such as insertional mutagenesis and ongoing expression of transgenes. Non-integrative methods, such as episomal vectors (oriP/EBNA1), Sendai virus-based RNA delivery, and synthetic mRNA transfection, have been developed to overcome these issues. These methods avoid permanent changes to the genome and are now preferred for generating iPSCs suitable for clinical and translational use (81–83). Among the many types of somatic cells that can be used to generate iPSCs, epithelial cells from the urinary tract are particularly appealing. Their collection is easy and non-invasive (84). Once created, iPSCs exhibit exceptional genetic flexibility. This allows for both directed differentiation with external stimuli, such as cytokines, growth factors, and morphogens, and precise genetic changes using advanced genome-engineering tools.

However, immunological compatibility remains a critical hurdle in clinical use. iPSCs intended for transplantation must be rendered hypoimmunogenic to minimize the risk of immune rejection (85). One strategy to address this challenge involves suppression of major histocompatibility complex (MHC) class I expression, specifically targeting β2-microglobulin (β2M), a key component of the MHC class I complex. In early approaches, a short hairpin RNA (shRNA) targeting β2M mRNA was delivered via a lentiviral vector system. Upon genomic integration, the vector constitutively expresses shRNA that mimics endogenous miRNA processing, leading to the sustained silencing of β2M (86, 87). Depending on the degree of sequence complementarity, the RNA-induced silencing complex (RISC) either degrades the β2M transcript or sterically blocks translation, thereby reducing cell surface expression of MHC class I molecules (88). RNA interference (RNAi)-based method has several advantages: it is stable over time due to continued intracellular expression, non-disruptive to the genome’s coding sequences, and yields immunologically ‘universal’ iPSCs suitable for a broad range of recipients. Alternatively, genome editing technologies have enabled permanent knockout of β2M. Targeted nucleases such as transcription activator-like effector nucleases (TALENs) have been used to disrupt exon 2 of the β2M gene, leading to functional abrogation of MHC class I presentation (5). More recently, CRISPR/Cas9 has emerged as a precise and efficient genome editing tool with improved specificity and reduced off-target activity, making it well-suited for clinical-grade iPSC engineering (13). Despite the substantial potential of these strategies, their clinical application requires thorough evaluation of potential risks. Genome engineering techniques may introduce off-target changes, structural variants, or copy-number alterations that could affect long-term genomic stability and therefore must be examined for unintended impacts on cellular fitness, clonal selection, or tumor-like immune evasion. Current regulatory frameworks for advanced therapy medicinal products (ATMPs) increasingly demand comprehensive genomic-integrity profiling, long-term *in vitro* passaging studies, and *in vivo* biodistribution and tumorigenicity assessments. Considering these factors may offer a realistic evaluation of the translational path for iPSC-derived megakaryocyte and platelet products (89–91).

An alternative approach to reducing immunogenicity in iPSC-based therapies involves the use of HLA-homozygous-induced pluripotent stem cell lines. Given the extreme polymorphism of human leukocyte antigens, achieving full histocompatibility between donor-derived iPSCs and unrelated recipients is inherently challenging. However, iPSC lines derived from individuals homozygous for the major HLA loci (HLA-A, -B, and -DR) offer a pragmatic solution. These lines can be selected or generated from donors with HLA haplotypes that are highly prevalent in a given population, thereby maximizing the likelihood of partial matching with multiple recipients. Notably, studies conducted in East Asian populations have demonstrated that a relatively limited number of HLA-homozygous iPSC lines could provide HLA-matched cell therapy options to over 70% of the population with minimal risk of acute rejection or graft-versus-host reactions (92). This strategy offers several advantages over genome-editing–based methods for immunoevasion. First, it circumvents the risks associated with genetic manipulation such as off-target effects or insertional mutagenesis. Secondly, HLA-homozygous iPSCs retain endogenous HLA class I expression, thus avoiding natural killer (NK) cell–mediated cytotoxicity, which typically targets cells lacking HLA (“missing-self” recognition). Third, these lines can be extensively characterized and qualified for clinical-grade use, forming the basis of universal donor iPSC banks. Such repositories, being developed in Japan and European countries, aim to provide immunologically compatible allogeneic cell products for various therapeutic applications, including hematopoietic, cardiological, and immunological indications (93, 94). Although there are challenges in identifying suitable homozygous somatic cell donors and HLA typing on a large scale, the HLA homozygosity-based approach allows for a scalable pathway for off-the-shelf iPSC-based therapies with reduced immunological barriers.

### 4.2 Differentiation protocols: cytokines, signaling, and culture systems

To initiate the differentiation of pluripotent stem cells into hematopoietic cells, it is essential to recreate the microenvironmental conditions and signals that regulate early embryonic hematopoiesis, while maintaining controlled culture conditions. The use of defined and xenogeneic-free reagents minimizes variability, which is critical for translational and clinical applications. Several differentiation protocols have been established to mimic spatiotemporal dynamics of early embryogenesis. Initial strategies focused on re-establishing the signaling environment of primitive streak and mesoderm formation, a critical step in which hematoendothelial precursors arise. For this purpose, BMP4 and WNT3a were used to induce the formation of a mesodermal bud with hemangioblastic potential, a structure capable of giving rise to both endothelial and hematopoietic progenitors (95).

In the developed differentiation schemes, the induction of hemogenic endothelium from pluripotent stem cells is typically achieved with a combination of BMP4, basic fibroblast growth factor (bFGF), and vascular endothelial growth factor (VEGF). Instead of direct use of WNT3a, many protocols rely on CHIR99021 (CHIR), a glycogen synthase kinase 3β (GSK-3β) inhibitor, to activate the canonical WNT signaling pathway. GSK-3β inhibits WNT/β-catenin signaling; thus, CHIR promotes the stabilization and nuclear translocation of β-catenin, a driver of mesodermal specification and hematopoietic potential (5, 96).

Exposure to hematopoietic cytokines such as stem cell factor (SCF), fms-like tyrosine kinase 3 ligand (Flt3L), and thrombopoietin (TPO) drives the transition of early mesodermal cells into hematopoietic progenitor populations. These progenitors subsequently generate primitive blood cell lineages such as early erythroid cells, megakaryocytes, and myeloid lineage cells (97). After hematopoietic identity is established, differentiation into mature cell types depends on precisely timed signals. During megakaryocyte development, this process involves a combination of cytokine-mediated signaling and physical factors that guide lineage commitment, cytoplasmic maturation, and the eventual production of platelets. SCF and TPO, or its small-molecule agonist TA-316, targeting the MPL receptor, play key roles during the early phase by promoting proliferation, survival, and expansion of megakaryocyte progenitors through activation of JAK/STAT and PI3K/AKT signaling cascades. Interleukin-11 (IL-11), particularly in combination with IL-6, drives differentiation by activating the STAT3 pathway and advancing progenitor cells toward a committed megakaryocytic state (98, 99). In the terminal stages of megakaryocyte differentiation, interleukin-1β (IL-1β) plays an important role by destabilizing the microtubule cytoskeleton and inducing membrane rupture, processes that facilitate proplatelet formation, and platelet shedding. Concurrently, the small-molecule inhibitor KP-457 has been shown to promote functional maturation by indirectly upregulating the CD42 complex via inhibition of ADAM17, a metalloproteinase involved in receptor shedding. In addition to these biochemical stimuli, the application of shear stress, which mimics the hemodynamic forces present in the vascular niche, serves as a crucial mechanical signal that drives cytoplasmic elongation and the final release of platelets. Together, these stimuli support the transition of induced pluripotent stem cells into mature. platelet-producing megakaryocytes *in vitro* (64, 100, 101). A summary of the cytokines and molecular mediators governing sequential progression from pluripotent stem cells to mature megakaryocytes is presented in **Table 1**.

**Table 1:** Overview of cytokines and signaling factors involved in monolayer differentiation from iPS cells to megakaryocytes.

Differentiation stage	Cytokine/factor	Function	Reference
Activation stage (2–3 days)	Wnt3A	WNT signaling is involved in early embryonic patterning, organogenesis, and tissue regeneration.	(102)
	CHIR99021	Selective GSK-3β inhibitor; promotes activation of the WNT/β-catenin signaling pathway.	(96)
	Activin A	A TGFβ superfamily ligand that promotes embryonic mesoderm formation via the SMAD2/3 signaling axis.	(103)
Mesoderm and endothelial induction (5–7 days)	BMP4	A TGFβ superfamily ligand that initiates SMAD1/5/8-mediated signaling and may also influence WNT and Notch signaling through pathway cross-regulation.	(104)
	bFGF	Essential for maintaining self-renewal and differentiation potential by activating the MAPK, PI3K/AKT, and JAK/STAT signaling pathways.	(105)
	VEGF	Promotes mesodermal specification and the generation of *in vitro* “pseudo-hemangioblasts” through activation of MAPK, PI3K/AKT, and other pathways shared with bFGF.	(106)
HSC/HPC induction (7–10 days)	SCF	Ligand for the c-Kit receptor tyrosine kinase; promotes proliferation, migration, survival, and differentiation of hematopoietic progenitors through activation of downstream MAPK, PI3K/AKT, and JAK/STAT pathways.	(98)
	IL-3	Facilitates hematopoietic lineage emergence by enhancing the endothelial-to-hematopoietic transition (EHT), thereby contributing to the specification and expansion of hematopoietic progenitor cells.	(107)
	IL-6	A pleiotropic cytokine that drives hematopoietic differentiation by engaging JAK/STAT, MAPK, and Notch signaling cascades.	(108)
	IL-11	A member of the IL-6 cytokine family that acts synergistically with IL-6 to promote the maturation of megakaryocyte progenitors through activation of the JAK/STAT3 signaling pathway.	(109)
	Flt3L	Essential for early hematopoietic progenitor maintenance; activates PI3K/AKT and ERK pathways via FLT3 receptor signaling, promoting cell proliferation, survival, and lineage potential.	(110)
	TPO	Ligand for the MPL receptor that promotes proliferation, survival, and differentiation of megakaryocytes and platelet precursors via JAK2–STAT3/5, PI3K/AKT, and RAS/MAPK pathways. Also supports hematopoietic stem cell expansion and stress-induced hematopoiesis.	(111)
Maturation stage (12–20 days)	KP-457	Small-molecule compound that indirectly enhances CD42 complex expression by inhibiting ADAM17, thereby promoting megakaryocyte maturation.	(112)
	IL-1β	Proinflammatory cytokine that promotes thrombogenesis by disrupting tubulin organization and inducing plasma membrane instability in megakaryocytes, leading to membrane rupture and proplatelet release.	(113)
	TA-316	A synthetic small-molecule agonist of the MPL receptor that mimics thrombopoietin activity. It promotes the proliferation, survival, and differentiation of megakaryocyte progenitors by activating downstream JAK2–STAT and PI3K/AKT signaling pathways.	(112)
	Shear stress	Biomechanical stimulus that promotes proplatelet elongation and platelet shedding by replicating hemodynamic forces present in the bloodstream.	(101)

Maintenance and hematopoietic differentiation of hESCs and iPSCs are frequently conducted on matrix-coated culture surfaces, which provide essential adhesive and biochemical cues in feeder-free systems. Substrates, such as Matrigel and Geltrex, derived from extracellular matrix proteins secreted by Engelbreth-Holm-Swarm (EHS) mouse sarcoma cells, contain laminin, collagen IV, entactin, and heparan sulfate proteoglycans. These substrates facilitate effective adhesion, proliferation, and differentiation of pluripotent stem cells. However, their use in clinical settings is constrained by their partly undefined formulation and nonhuman origin. To address these drawbacks, fully defined xeno-free substrates have been developed. Among them, recombinant human laminins 511 and 521 have shown strong potential to preserve stem cell pluripotency and promote hematopoietic differentiation of hESCs and iPSCs within GMP-compliant systems. Their consistency and compliance with regulatory requirements make them well-suited for clinical-grade applications (114, 115).

An interesting approach to obtain megakaryocytes in large quantities is the immortalization of iPSC-derived megakaryocyte precursors, which allows for the production of inducible megakaryocyte cell lines (imMKC). Immortalization has been achieved by the retroviral transduction of hematopoietic progenitor cells with expression constructs involved in anti-apoptotic and self-renewal-promoting activities, such as *BMI1*, *BCL-XL*, and *c-MYC* (116). The expression of these genes is regulated by the Tet-On system, which allows the precise control of proliferation and differentiation under the influence of doxycycline. In the presence of the inducer, the imMKCs remained in an undifferentiated progenitor state. After doxycycline withdrawal, the cells exit the progenitor stage and mature into megakaryocytes capable of producing platelets. Cell functionality is enhanced by physical stimuli such as shear stress and turbulent energy. To eliminate the risk of carcinogenesis, the final product is irradiated, which stops replication but preserves its function (72, 117).

Differentiation protocols may differ in their ultimate goals or culture strategies, but the transition from iPSCs to hematopoietic cells proceeds via the sequential induction of mesodermal and hematoendothelial intermediates. The evaluation of the efficiency of this process requires phenotypic analysis of the resulting cell population. Flow cytometry remains the primary analytical tool for this purpose as it enables the detection of cell surface markers. This allows for the identification of mature hematopoietic lineages and the tracking of the dynamics of early progenitor emergence. Mature megakaryocytes are defined by the co-expression of CD41 (integrin αIIb) and CD42b (GPIbα), erythrocyte cells by CD41 and CD235a, whereas myeloid cells are usually positive for CD45, CD18, and CD33 (97).

Megakaryocyte and erythrocyte progenitors (MEPs) exhibit a characteristic CD235^+^CD31^+^ phenotype, reflecting their hemogenic endothelial origin and bipotential fate. More lineage-restricted megakaryocyte progenitors (MKPs) express CD41 and CD61 (β3 integrin), indicating progression toward the megakaryocyte lineage (21). These immunophenotypic markers are used to monitor the timing and efficiency of differentiation as well as to quantify lineage bias during differentiation. By mapping these populations at defined time points, flow cytometry enables optimization of the set of cytokines, culture substrates, and chemical compounds that modulate certain signaling pathways.

While immunophenotyping provides essential information about lineage identity and maturation status, the success of any differentiation protocol ultimately depends on functional assays performed on the platelets produced *in vitro*. Key evaluations involve examining activation and aggregation following stimulation with agonists like TRAP, ADP, and thrombin, alongside analyzing surface marker expression such as PAC-1 and P-selectin. Adhesion assays to fibrinogen and von Willebrand factor are also conducted. Morphological assessment is crucial, including both quantitative and qualitative analysis of platelet granules using confocal and transmission electron microscopy, as well as evaluating cytoskeletal changes post-activation. Additionally, monitoring hematological parameters like mean platelet volume (MPV) and the immature platelet fraction (IPF) is essential. The final confirmation comes from functional testing *in vivo* using animal models, which includes assessing hemostasis restoration and platelet survival (72, 117, 118).

## 5 Clinical applications and translational challenges

### 5.1 Limitations of donor-derived platelet transfusions

Progress in the understanding of hematopoiesis at the molecular level, along with new *in vitro* protocols, has enabled platelet production as a potential alternative to conventional preparations. This approach offers improved safety through greater control and standardization. iPS cells represent a virtually unlimited and ethically noncontentious source for this purpose. Their amenability to precise genetic modification using tools such as CRISPR and its recent advancements (e.g., Prime Editing) opens the possibility of tailoring cell products for patients who no longer respond to donor-derived allogeneic platelets. However, this strategy presents several challenges. In particular, complete ablation of HLA class I molecules, used to reduce immune rejection, may paradoxically increase NK-mediated cytotoxicity, thereby limiting therapeutic efficacy (116). To reduce this risk, only a partial suppression of β2-microglobulin expression is recommended. Supporting this concept, a study using mouse fibroblasts with progressive knockout of HLA class I showed that NK cell-mediated lysis was significantly increased when the knockout efficiency exceeded 90% (119).

Nonetheless, strategies that rely solely on eliminating immunogenicity through β2-microglobulin inactivation remain incomplete. Although this approach effectively prevents allorecognition by CD8^+^ T cells, it induces strong NK cell activation. Such modifications limit the persistence and immunological safety of pluripotent-derived cells within the recipient’s immune environment (120). For these reasons, current approaches focus on reconstructing a minimal, tolerogenic HLA phenotype that constrains both T lymphocytes and NK cells. One of the most promising directions is the introduction of non-polymorphic HLA molecules with immunoregulatory functions, such as HLA-E or HLA-G. These molecules, by stabilizing interactions with NKG2A/CD94 receptors and selected KIR receptors, provide inhibitory signals sufficient to avoid NK cytotoxicity, while lacking epitopes capable of eliciting alloantibodies. Importantly, their expression does not restore the high level of antigenicity characteristic of classical HLA-A and HLA-B (120–122). In recent years, it has also been demonstrated that inhibitory signals generated by HLA-E and HLA-G can, under inflammatory conditions, compensate for the partial loss of classical HLA, limiting both the primary activation of NK cells and the subsequent amplification of the effector response induced by pro-inflammatory cytokines. Additionally, the non-polymorphic nature of these molecules reduces the risk of inducing specific CD8^+^ clones and the *de novo* formation of anti-HLA antibodies, which is important for maintaining cell functionality after repeated administration (123–125).

Concurrently, strategies are being developed to modulate T cell activity through the expression of immune checkpoint ligands, such as PD-L1. Its overexpression reduces signaling through PD-1 on activated T lymphocytes, decreasing cytotoxicity against iPSC-derived cells even when the expression of classical HLA molecules is reduced (125, 126). In some approaches, additional tolerogenic pathways like CTLA-4/CD80-CD86 or TIM-3/Gal-9, are modulated simultaneously, further enhancing peripheral T cell tolerance and limiting the recruitment of antigen-presenting cells (127–129). These strategies are often combined with maintaining limited expression of selected HLA-C alleles or with the modulation of phagocytosis-regulating molecules, such as CD47, which further decreases the risk of cell removal by macrophages and the secondary activation of T cell responses. A high level of CD47 is particularly important, as it helps avoid both classical phagocytosis and inflammation-induced opsonin-driven clearance, which can destabilize even partially tolerogenic HLA configurations (130–132).

The primary goal of the above strategies is to achieve a phenotype that simultaneously minimizes the adaptive immune response and preserves key inhibitory signals of the innate immune system, which are essential for avoiding activation of NK cells and phagocytes. In the context of megakaryocytes generated from pluripotent stem cells, this is especially important because the final transfusion product, the platelet, is devoid of a genome, and therefore any immunological modification must be introduced at the precursor cell stage. Designed modifications can thus create stable and functionally “universal” platelet producing lines, capable of bypassing classic alloimmune barriers, including antibody-dependent HLA refractoriness.

*In vitro* platelet production faces challenges regarding efficiency and the need to optimize the process. Without the use of genetic modifications, current protocols yield disappointing results, with a maximum of approximately 30 platelets per megakaryocyte differentiated from iPSCs (133). In contrast, mature megakaryocytes in adult organisms produce up to 2,000 platelets. It is estimated that approximately 100 million mature megakaryocytes are required to generate a single therapeutic dose of platelet concentrate, corresponding to approximately 2×10^11^ platelets (134). This disparity suggests that megakaryocytes obtained through *in vitro* differentiation more closely resemble their primitive counterparts and lack the full maturation potential.

Comparable functional immaturity is also observed in neonatal platelets, which show impaired aggregation and secretion due to impaired intracellular signaling. These limitations have prompted the consideration of alternative strategies such as obtaining megakaryocytes from the bone marrow. However, this approach is highly invasive and inefficient; the concentration of megakaryocytes in the bone marrow ranges from 50 to 150 cells/µL, representing less than 0.1% of the nucleated cells in the bone marrow (135). Furthermore, these cells have limited replicative capacity and are highly susceptible to stress-induced apoptosis *in vitro*. Despite these obstacles, advances in culture conditions have indicated that many current barriers to scalable *in vitro* platelet production may eventually be overcome.

### 5.2 iPSC-derived platelets in clinical trials: case study of iPLAT1

A significant milestone in platelet bioengineering was achieved with the launch of the iPLAT1 clinical trial, which provided a proof of concept for autologous production of functional platelets *in vitro* from genetically modified cells. In this strategy, induced pluripotent stem cell (iPSC)-derived megakaryocyte progenitor cells are genetically immortalized to permit sustained expansion while preserving their differentiation potential under tightly controlled culture conditions. Maturation into platelet-producing megakaryocytes was conducted within a turbulent flow bioreactor system designed to mimic the biomechanical environment of the bone marrow sinusoids. Such dynamic forces are essential for promoting proplatelet extension and cytoplasmic fragmentation, hallmarks of physiological thrombopoiesis. The bioreactor achieved a platelet yield of 70–80 per megakaryocyte, representing a more than two-fold improvement over the conventional static culture systems. This corresponded to a total output of approximately 100 billion platelets per production cycle in an 8-liter system, approaching the therapeutic dose required for clinical application (136). To eliminate the risk of tumor formation from residual progenitor cells, the final platelet product was exposed to 25 Gy gamma irradiation, which was sufficient to prevent cellular replication without compromising the platelet structure or function. The treated platelets were then administered to a patient with aplastic anemia in a dose-escalation regimen consisting of autologous transfusions of 1 × 10¹^0^, 3 × 10¹^0^, and 1 × 10¹¹ platelets, delivered at three-month intervals.

The 360-day observation period revealed no signs of toxicity related to infusion, immune complications, or indications of uncontrolled cell growth, thus supporting the safety profile of the product. Flow cytometry and morphological assessments indicated minor differences between the transfused and native platelets, with the former displaying slightly increased size (by approximately 1–1.5 µm) and altered granularity. These differences likely stem from incomplete cytoskeletal maturation and are characteristic of platelets generated from early-stage hematopoiesis or neonatal sources. While preclinical evaluations confirmed satisfactory hemostatic function, further studies are necessary to assess their *in vivo* survival, integration into the recipient’s coagulation network, and functional performance during bleeding or thrombotic events.

The iPLAT1 study supported the idea that personalized platelet therapy created in the lab could be technically feasible and safe for patients. This new approach may be a good option for patients who experience donor platelet alloimmunization, have trouble with repeated transfusions, or require HLA-matched products. In the long run, these technologies could be used in point-of-care manufacturing systems, allowing on-demand delivery of platelets (126). More research is needed to improve megakaryocyte maturation, boost proplatelet yield, and enhance post-transfusion function. Strategies currently under investigation include modulation of cytoskeletal regulators (e.g., tubulin isoforms), introduction of synthetic scaffolds to mimic bone marrow niches, and refinement of shear-stress application dynamics to more precisely guide the thrombopoietic process (137–139).

### 5.3 Platelets as drug delivery platforms: potential in hemophilia A

One direction for the application of *in vitro*-generated platelets is their use as therapeutic vectors, particularly in disorders associated with coagulation factor deficiency. In hemophilia A, which is characterized by a lack of coagulation factor VIII (FVIII), therapeutic measures include the transfusion of plasma or FVIII concentrates. However, 30% of patients with severe disease develop neutralizing antibodies (inhibitors) against exogenous FVIII, which reduces treatment efficacy. In this context, alternative therapeutic strategies focus on directing FVIII expression to megakaryocytes, enabling its localization to platelet α-granules. These platelet-derived FVIII (pFVIII) molecules are stored intracellularly and released locally at sites of vascular injury where they can act independently of circulating inhibitors. Studies using a transgenic mouse model (pFVIII/FVIII^null^) have shown that pFVIII effectively corrects coagulation deficits even in the presence of high titers of circulating anti-FVIII antibodies. In a FeCl_3_-induced carotid artery injury model, pFVIII provided protection at inhibitor levels (measured in Bethesda units per milliliter, BU/mL) six times higher than plasma-derived FVIII delivering a comparable antigen level (140). The advantage of this strategy lies in the limited exposure of FVIII to circulating antibodies; pFVIII remains protected within platelets until its activation and degranulation. It was also observed that a pFVIII level corresponding to only ~9% of the plasma antigen correction was sufficient to achieve a therapeutic effect comparable to that of plasma FVIII infusion in the range of 25–50%. Furthermore, antibody-mediated platelet reduction was not observed, thereby confirming the safety of this approach (140).

From a clinical perspective, iPSC-derived platelets carrying factor VIII could be used as targeted drug carriers, releasing the factor only at the sites of vascular injury. This approach would reduce the need for high circulating FVIII doses and lowers the risk of immunization. Furthermore, owing to the possibility of genetically modifying iPSCs prior to their differentiation into megakaryocytes, it is conceivable to design FVIII constructs resistant to antibody recognition, for example, by altering epitopes vulnerable to neutralization without compromising the functionality of the factor.

Recent efforts have focused on improving the performance of this platform. The challenges include increasing the number of platelets generated, improving the efficiency of FVIII incorporation into α-granules, and improving the conditions for megakaryocyte maturation. These include the use of flow-controlled bioreactors and artificial hematopoietic niches (141, 142).

## 6 Conclusions and future directions

Since the beginning of medicine, the blood and its components have become crucial in both clinical practice and biomedical research. Significant progress has been made in the development of platelet preparations that prioritize patient safety and therapeutic efficacy. Although the risks associated with the transfusion of donated blood products have decreased significantly in recent decades, a number of immunological and logistical challenges remain. These include alloimmunization, transfusion resistance, pathogen transmission, and limited donor availability, all of which are exacerbated by the aging population in high-income countries.

In this context, stem cell-based interventions and *in vitro* generation of blood cells offer promising alternatives to the challenges of transfusion medicine. The use of iPSCs provides a potentially unlimited source of progenitors for platelet production, which allows autologous or HLA-matched therapies. However, current differentiation protocols are still flawed, especially when it comes to producing large amounts of mature and physiologically capable platelets. Problems with megakaryocyte maturation, proplatelet formation, and platelet efficiency hinder the practical use of *in vitro*-derived products. These issues emphasize the need for further research on the molecular and cellular processes that control human blood cell formation. Creating a functional and universally usable platelet concentrate that does not rely on donor blood is a major goal of transfusion medicine and regenerative therapy. Further advances in bioprocessing, genome engineering, and recreation of bone marrow-like microenvironments are expected to accelerate the clinical adoption of *in vitro-*generated platelets as next-generation therapeutic platforms.

## Glossary

AGM: Aorta-gonad-mesonephros
ATMP: advanced therapy medicinal product
bFGF: Basic fibroblast growth factor
BMP4: Bone morphogenetic protein 4
BU: Bethesda units
CD34: Cluster of differentiation 34
CD41: Cluster of differentiation 41
CD42: Cluster of differentiation 42
CD45: Cluster of differentiation 45
CHIR99021: Glycogen synthase kinase-3 (GSK-3) inhibitor
CLP: Common lymphoid progenitor
CMP: Common myeloid progenitor
CRISPR: Clustered regularly interspaced short palindromic repeats
EMP: Erythro–myeloid progenitor
EryD: Definitive erythroid progenitor
EryP: Primitive erythroid progenitor
ESC: Embryonic stem cell
FeCl₃: Ferric chloride
FVIII: Factor VIII
GATA1: GATA-binding protein 1
GMP: Good manufacturing practice
HLA: Human leukocyte antigen
HPA: human platelet antigens
HPSC: Hematopoietic stem and progenitor cells
HSC: Hematopoietic stem cell
imMKC: immortalized megakaryocyte cell line
IPF: immature platelet fraction
iPSC: Induced pluripotent stem cell
LMPP: Lymphoid-primed multipotent progenitor
LT-HSC: Long-term hematopoietic stem cell
MEP: Megakaryocyte–erythroid progenitor
MHC: Major histocompatibility complex
MK: Megakaryocyte
MKP/MKPs: Megakaryocyte progenitor(s)
MP: Megakaryocyte progenitor
MP: Megakaryocytic progenitor
MPP2: Multipotent progenitor 2, progenitor with megakaryocyte–erythroid bias
MPV: mean platelet volume
NK: Natural killer (cell)
p-MEP: Primitive megakaryocyte–erythroid progenitor
PB: Peripheral blood
PDGF: platelet-derived growth
PF4: platelet factor 4
pFVIII: Platelet-targeted factor VIII
pMK: Primitive megakaryocyte
pMP: Primitive myeloid progenitor
RISC: RNA-induced silencing complex
RNAi: RNA interference
shRNA: Short hairpin RNA
ST-HSC/MPP1: Short-term hematopoietic stem cell/multipotent progenitor 1
TALEN: Transcription activator-like effector nuclease
TGF-β: transforming growth factor-beta
TPO: Thrombopoietin
VEGF: Vascular endothelial growth factor
vWF: von Willebrand factor.
